# Acute kidney injury and mild therapeutic hypothermia in patients after cardiopulmonary resuscitation - a post hoc analysis of a prospective observational trial

**DOI:** 10.1186/s13054-018-2061-6

**Published:** 2018-06-08

**Authors:** Julia Hasslacher, Fabian Barbieri, Ulrich Harler, Hanno Ulmer, Lui G. Forni, Romuald Bellmann, Michael Joannidis

**Affiliations:** 1 Division of Intensive Care and Emergency Medicine, Department of Internal Medicine, Medical University Innsbruck, Anichstr. 35, 6020 Innsbruck, Austria; 2 Department of Medical Statistics, Informatics and Health Economics, Medical University Innsbruck, Schöpfstr. 41/1, 6020 Innsbruck, Austria; 3 Intensive Care Unit, Royal Surrey County Hospital NHS Foundation Trust, Egerton Road, Guildford, UK; 4 Department of Clinical & Experimental Medicine, Faculty of Health Sciences, University of Surrey, Guildford, UK

**Keywords:** Acute kidney injury, Mild therapeutic hypothermia, Cardiopulmonary resuscitation, Neurological outcome, Cystatin C, Creatinine

## Abstract

**Background:**

The aim of this study was to investigate the influence of mild therapeutic hypothermia (MTH) on the incidence of and recovery from acute kidney injury (AKI).

**Methods:**

Patients who had undergone successful cardiopulmonary resuscitation (CPR) were included. Serum creatinine and cystatin C were measured at baseline, daily up to 5 days and at ICU discharge. AKI was defined by the Kidney Disease Improving Global Outcomes (KDIGO) criteria. MTH was applied for 24 h targeting a temperature of 33 °C. Neurological outcome was assessed with the Cerebral Performance Categories score at hospital discharge.

**Results:**

126 patients were included in the study; 73 patients (58%) developed AKI. Patients treated with MTH had a significantly lower incidence of AKI as compared to normothermia (NT) (44 vs. 69%; *p* = 0.004). Patients with less favourable neurological outcomes had a significantly higher rate of AKI, although when treated with MTH the occurrence of AKI was reduced (50 vs. 80%; *p* = 0.017). Furthermore, MTH treatment was accompanied by significantly lower creatinine levels on day 0–1 and at ICU discharge (day 0: 1.12 (0.90–1.29) vs. 1.29 (1.00–1.52) mg/dl; *p* = 0.016) and lower cystatin C levels on day 0–3 and at ICU discharge (day 0: 0.88 (0.77–1.10) vs. 1.29 (1.06–2.16) mg/l; *p* < 0.001).

**Conclusions:**

Mild therapeutic hypothermia seems to have a protective effect against the development of AKI and on renal recovery. This may be less pronounced in patients with a favourable neurological outcome.

**Electronic supplementary material:**

The online version of this article (10.1186/s13054-018-2061-6) contains supplementary material, which is available to authorized users.

## Background

The development of acute kidney injury (AKI) is associated with mortality rates ranging from 30 to 70% although this depends on aetiology. AKI frequently complicates out-of-hospital cardiac arrest (OHCA) with reported rates between 12 and 40% [1, 2]. Moreover, after OHCA severe forms of AKI are more common (AKI stage 3 according to the Acute Kidney Injury Network (AKIN) classification) and are associated with poorer outcome [3]. Following successful resuscitation the return of spontaneous circulation (ROSC) heralds a complex pathophysiological process, the “post-cardiac arrest syndrome” which comprises:

1. Post-cardiac arrest brain injury
2. Post-cardiac arrest myocardial dysfunction
3. Systemic ischaemia/reperfusion response
4. Persistent precipitating pathology [4, 5]

In particular, reperfusion injury after global ischaemia leads to systemic inflammatory response syndrome with consecutive tissue damage, also affecting the kidneys. In a murine model of cardiopulmonary resuscitation (CPR) there was significant infiltration of leukocytes in the kidneys and histomorphologic signs of AKI accompanied by elevated creatinine and urea levels after cardiac arrest (CA) [6]. A characteristic morphological injury pattern in renal tissue, such as interstitial edema, renal tubular necrosis and inflammatory cell infiltration, and elevated AKI biomarkers such as neutrophil gelatinase-associated lipocalin (NGAL) and cystatin C were observed in a swine model of AKI after CA with ventricular fibrillation and asphyxiation [7].

Mild therapeutic hypothermia (MTH) is applied after CPR to attenuate the consequences of post cardiac arrest syndrome [4], but little is known about its influence on renal function, renal pathophysiological processes and the incidence of AKI. A previous study investigating renal function after CPR reported delayed improvement of renal function as determined by 4-h creatinine clearances during treatment with MTH, but there were no significant differences in serum creatinine levels or urinary output when compared to treatment with normothermia [8]. A meta-analysis of randomized controlled trials did not demonstrate significant association between hypothermia and the risk of AKI or the need for dialysis, although there was positive correlation between lower target temperature and rates of AKI [9]. Unfortunately these studies had only small sample sizes and were performed before the establishment of the current Kidney Disease Improving Global Outcomes (KDIGO) AKI classification.

The aim of our study was to investigate the incidence of AKI in patients after successful CPR, using the KDIGO classification [10] and to evaluate the influence of MTH on AKI development and other indices of renal function.

## Methods

This is a secondary analysis of our previously published study investigating neurological outcome prediction of secretoneurin after successful CPR [11]. This prospective observational single-centre trial included consecutive adult patients (age ≥ 18 years) admitted to the medical intensive care unit (ICU) of the University Hospital of Innsbruck from September 2008 to April 2013 after successful CPR. Neuroendocrine tumour, stroke, intracranial haemorrhage or trauma as the cause of cardiac arrest or life expectancy of less than 24 h as determined by the treating physicians were exclusion criteria.

Serum creatinine was measured at baseline, daily up to 5 days (day 0–4) and at ICU discharge. Serum cystatin C was measured daily from day 0−4 and at ICU discharge. The baseline creatinine values were determined based on the following algorithm:

1. The median of all values available in the time range between 6 months prior to admission and ending 6 days prior to enrolment was taken if at least five values were available or if the number of values available exceed the number available in the time range starting 5 days prior to enrolment and ending at enrolment or
2. Nadir value in the time range starting 5 days prior to enrolment and ending at enrolment, if at least one value was available or
3. Enrolment value, if the value is in normal range or
4. Value calculated by the modification of diet in renal disease (MDRD) formula [12]

The occurrence of AKI was determined according to the KDIGO guidelines, based on serum creatinine values [10]. We could not apply urinary output criteria properly, because urinary output had not been recorded at 6-h intervals. We also documented the patients that received renal replacement therapy (RRT). Patients were excluded from the study if chronic renal insufficiency (chronic kidney disease (CKD) > stage G3) was reported in the medical history and no baseline creatinine measurement was available. Patients receiving RRT were excluded from any further calculations involving creatinine and cystatin C values.

Estimated glomerular filtration rate (eGFR) was calculated from creatinine and cystatin C values using the Chronic Kidney Disease Epidemiology Collaboration (CKD-EPI) formula respectively [13] at ICU discharge. Sequential Organ Failure Assessment (SOFA), Acute Physiology and Chronic Health Evaluation II (APACHE II) scores and the use of catecholamines were documented at the day of ICU admission. Cardiac arrest data such as the rate of bystander resuscitation, time to ROSC and first monitored rhythm were collected from the emergency or heart alarm protocol according to the Utstein style [14]. Furthermore, it was documented if patients underwent MTH using an intravascular cooling device targeting a core body temperature of 33 °C (measured in the urinary bladder) for 24 h. According to the guidelines at study initiation MTH was routinely applied to comatose patients with an initially shockable rhythm that had received advanced life support within 15 min and showed a ROSC within 60 min after collapse. After modification of the European Society of Cardiology (ESC) guidelines in 2010, MTH was also applied to comatose patients with an initially non-shockable rhythm, if the event was observed and time to ROSC was less than 25 min [15]. Neurological outcome was determined by the Cerebral Performance Categories Scale (CPC) immediately before discharge either from hospital or a long-term care facility, following a standardized protocol [11].

The study protocol was approved by the Ethics Committee of the Medical University of Innsbruck (protocol number UN3493 272/4.31). Written informed consent was obtained from next of kin or retrospectively from patients who recovered.

### Statistical analysis

Categorical data are given as counts and percentages, continuous data as means and standard deviations or medians with interquartile ranges. Normal distribution of continuous data was checked by the Kolmogorov-Smirnov test. As serum creatinine, cystatin C and most other variables were not normally distributed, the Mann-Whitney U test and the chi-square test were used for univariate comparison of categorical variables of neurological outcome, normothermia (NT) and MTH treatment. A logistic regression model was used to assess whether MTH treatment is protective against AKI after adjustment for clinical relevant variables such as SOFA score and time to ROSC. The accuracy of serum creatinine and cystatin C levels measured at day 0 in predicting AKI and neurological outcome was evaluated by receiver operating characteristic (ROC) analysis. Mixed effects model analysis was applied to evaluate the effect of MTH on creatinine and cystatin C levels (day 0–4). For this analysis, creatinine and cystatin C were logarithmically transformed to achieve normally distributed values. *P* values < 0.05 were considered statistically significant. IBM SPSS Statistics (IBM Corp. Released 2012. IBM SPSS (Statistics for Windows, Version 21.0. Armonk, NY, USA: IBM Corp.) was used to analyse data.

## Results

### Patient characteristics

There were 126 patients included in this analysis [11] (see Additional file 1): 55 patients were treated with MTH. The mean duration of MTH at the target temperature of 33 °C was 23.5 h (IQR 2.5) (3 h (IQR 2.0) during cooling down, 13 h (IQR 3.0) during rewarming and 34 h (IQR 23) during NT).

Patients treated with MTH were significantly younger (60 vs. 67 years; *p* = 0.007), had a lower rate of in-hospital cardiac arrest (4 vs. 17%; *p* = 0.019), a higher rate of a shockable first monitored rhythm (87 vs. 38%; *p* = 0.0001), a higher rate of vasopressor requirement (95 vs. 68%; *p* = 0.0001) and a lower APACHE II score (23 vs. 26; *p* = 0.0001) compared to those patients treated with NT (see Additional file 2). A total of 64 of 126 patients (50.8%) had a poor neurological outcome (CPC 3–5). Length of stay in the ICU (ICU-LOS) was 6 days (IQR 8). In patients with a favourable neurological outcome the length of stay was longer (8 days (IQR 9) vs. 4 days (IQR 6)).

### Acute kidney injury

A total of 73 of 126 patients (58%) developed AKI; 29 (40%) of them had AKI stage 3 including 20 (16%) patients who received RRT. Patients who developed AKI differed significantly by age, rate of bystander-initiated CPR, time to ROSC, poor neurological outcome, shockable first monitored rhythm, SOFA and APACHE II score at admission and MTH treatment as compared to patients without AKI (Table 1).

**Table 1:** Patients characteristics in patients with or without acute kidney injury (AKI)

	No AKI (*n* = 53)	AKI (*n* = 73)	*p* value
Median age (IQR)	58 (18)	68 (20)	0.001
Female, *n* (%)	18 (33)	15 (21)	ns
Bystander-initiated CPR, *n* (%)	43 (81)	41 (56)	0.003
Time to ROSC > 20 min, *n* (%)	22 (42)	49 (67)	0.004
Cardiac arrest in hospital, *n* (%)	5 (9)	9 (12)	ns
Poor neurological outcome, *n* (%)	19 (36)	45 (62)	0.004
Favourable neurological outcome, *n* (%)	34 (64)	28 (38)	0.004
Shockable first monitored rhythm, *n* (%)	39 (74)	36 (49)	0.007
Catecholamines on admission, *n* (%)	39(74)	61 (84)	ns
Baseline creatinine, MV ± SD (mg/dl)	0.95 ± 0.21	1.07 ± 0.97	ns
SOFA score, median (IQR)	9 (3)	11 (3)	0.0001
APACHE II score, median (IQR)	23 (6)	27 (7)	0.0001
MTH, *n* (%)	31 (58)	24 (33)	0.004

*Abbreviations: CPR* cardiopulmonary resuscitation, *ROSC* return of spontaneous circulation, *MV* mean value, *SOFA* Sequential Organ Failure Assessment, *APACHE* Acute Physiology and Chronic Health Evaluation, *MTH* mild therapeutic hypothermia, *ns* not significant

**Fig. 1:**
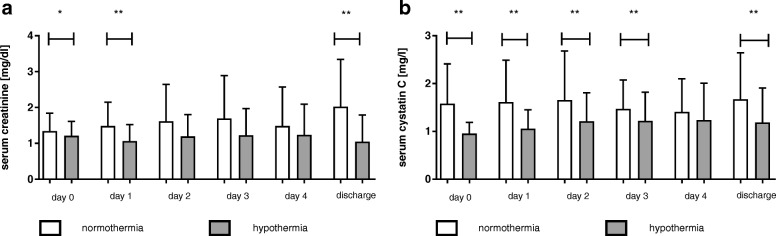
Serum creatinine and cystatin C in patients treated with mild therapeutic hypothermia or normothermia. All patients: serum creatinine (mg/dl) (**a**) and serum cystatin C (mg/l) (**b**) (mean and standard deviation) at day 0–4 and ICU discharge in patients treated with mild therapeutic hypothermia or normothermia; *p < 0.05; **p < 0.01

Patients treated with MTH (versus NT) had a significantly lower incidence of AKI (44 vs. 69%; *p* = 0.004) and a trend towards a reduced need for RRT (11 vs. 20%; *p* = 0.201) (Table 2). Correspondingly, patients treated with MTH had significantly lower creatinine levels on day 0 (1.12 (0.90–1.29) vs. 1.29 (1.00–1.52) mg/dl; *p* = 0.016), day 1 and at ICU discharge (0.86 (0.66–1.02) vs. 1.14 (0.82–2.03) mg/dl; *p* = 0.001) and lower cystatin C levels on day 0 (0.88 (0.77–1.10) vs. 1.29 (1.06–2.16) mg/l; *p* < 0.001), day 1–3 and ICU discharge (1.06 (0.88–1.38) vs. 1.66 (1.07–2.57) mg/l; *p* = 0.0001) as compared to patients treated with NT (Fig. 1).

**Table 2:** Number and percentages of patients with RRT and AKI according to outcome and targeted temperature management

A			
All patients (*n* = 126)	Normothermia (*n* = 71)	Hypothermia (*n* = 55)	*p* value
AKI, *n* (%)	49 (69)	24 (44)	0.004
AKI 1, *n* (%)	21 (30)	11 (20)	
AKI 2, *n* (%)	8 (11)	4 (7)	
AKI 3, *n* (%)	20 (28)	9 (16)	
RRT, *n* (%)	14 (20)	6 (11)	ns
B			
Favourable outcome (*n* = 62)	Normothermia (*n* = 27)	Hypothermia (*n* = 35)	*p* value
AKI, *n* (%)	14 (52)	14 (40)	ns
AKI 1, *n* (%)	5 (19)	8 (23)	
AKI 2, *n* (%)	2 (7)	3 (9)	
AKI 3, *n* (%)	7 (26)	3 (9)	
RRT, *n* (%)	5 (19)	2 (6)	ns
C			
Poor outcome (*n* = 64)	Normothermia (*n* = 44)	Hypothermia (*n* = 20)	*p* value
AKI, *n* (%)	35 (80)	10 (50)	0.017
AKI 1, *n* (%)	16 (36)	3 (15)	
AKI 2, *n* (%)	6 (14)	1 (5)	
AKI 3, *n* (%)	13 (30)	6 (30)	
RRT, *n* (%)	9 (21)	4 (20)	ns

Number (and percentages) of patients with continuous renal replacement therapy (RRT) and Acute kidney injury (AKI) stage 1–3 and only stage 3 (Kidney Disease Improving Global Outcomes (KDIGO)) in all patients (A), patients with good (B) and poor (C) neurological outcome treated with mild therapeutic hypothermia or normothermia

*ns* not significant

**Fig. 2:**
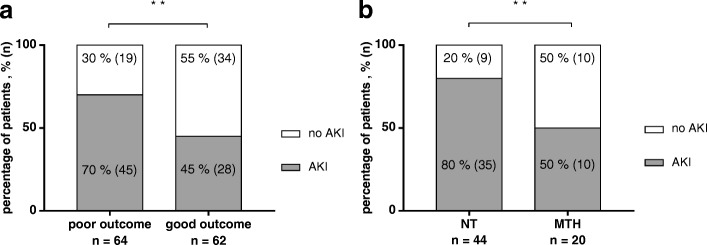
Acute kidney injury (AKI) after cardiopulmonary resuscitation (CPR). **a** Incidence of AKI in patients with good versus poor neurological outcome. All patients: percentage of patients developing AKI or no AKI in each subgroup with good (*n* = 64) or poor neurological outcome (*n* = 62); **p < 0.01. **b** Incidence of AKI in patients with poor neurological outcome treated with normothermia (NT) versus mild therapeutic hypothermia (MTH). Patients with poor neurological outcome: percentage of patients developing AKI or no AKI when treated with MTH (*n* = 20) or NT (*n* = 44); **p < 0.01

The subgroup of patients with poor neurological outcome had a significantly higher rate of AKI compared to patients with favourable neurological outcome (70 vs. 45%; *p* = 0.004) (Fig. 2). Furthermore they had significantly higher creatinine and cystatin C levels at several time points during the observation period (see Additional file 3).

In patients with a poor neurological outcome, further analysis revealed a lower incidence of AKI in patients treated with MTH as compared to NT (50 vs. 80%; *p* = 0.017) (Table 2, Fig. 2). Correspondingly, these patients had lower creatinine levels at ICU discharge and significantly lower cystatin C levels on day 0–1 (day 0: 0.96 (0.83–1.30 vs. 1.44 (1.17–2.29) mg/l; *p* = 0.01) and at ICU discharge (1.13 (0.93–1.38 vs. 1.68 (1.17–2.48) mg/l; *p* = 0.027) if treated with MTH (versus NT).

Patients with a favourable neurological outcome followed a similar, but non-significant trend with lower rates of AKI and RRT with MTH treatment (Table 2). Furthermore, they had significantly lower creatinine on day 1 (0.82 (0.67–1.02) vs. 1.03 (0.83–1.38) mg/dl; *p* = 0.049) and cystatin C on day 0 (0.87 (0.76–1.06) vs. 1.14 (0.90–1.50) mg/l; *p* = 0.049) and day 1 (0.85 (0.73–1.23) vs. 1.17 (0.91–1.69) mg/l; *p* = 0.023) if treated with MTH (versus NT).

### Renal recovery

Renal function at ICU discharge was defined by eGFR, which was significantly higher in patients treated with MTH versus NT (eGFR creatinine, 83 (44) vs. 53 (55) ml/min/1.73 m^2^; *p* = 0.0001; eGFR cystatin C, 71 (46) vs. 38 (51) ml/min/1.73 m^2^; *p* = 0.0001) (see Additional file 4). Furthermore we evaluated the need for RRT at hospital discharge: 20 patients received RRT, 11 of whom died in the ICU; 9 patients had need for RRT after CPR on the ICU, but none of them had further requirement of RRT at hospital discharge, which was after a median of 38 (27–66) days; 7 of them had a good neurological outcome.

### Factors influencing occurrence of AKI and serum levels of creatinine and cystatin C

In multivariate analysis mild therapeutic hypothermia reduced the risk of AKI and the SOFA score at admission was the significant factor for the development of AKI (Table 3). In the mixed model analysis we observed a significant influence of MTH on creatinine and cystatin C levels in the whole study population (*p* = 0.012 and *p* < 0.0001, respectively).

**Table 3:** Logistic regression analysis for the development of AKI

	Odds ratio (95% CI)	*p* value
Mild therapeutic hypothermia	0.424 (0.187–0.962)	0.040
SOFA score at admission	1.486 (1.211–1.824)	0.0001
Time to ROSC > 20 min	1.361 (0.577–3.209)	0.481

*AKI* acute kidney injury, *SOFA* Sequential Organ Failure Assessment, *ROSC* return of spontaneous circulation

In the subgroups we detected a statistically significant effect of MTH on cystatin C levels in patients with good neurological outcome (*p* < 0.0001) and on creatinine (*p* = 0.022) and cystatin C levels (*p* < 0.0001) in patients with poor neurological outcome.

### Prediction of AKI by creatinine and cystatin C

Serum creatinine values on admission reliably predicted the development of AKI, with an AUC of 0.873 (0.813–0.933). Similarly, for cystatin C levels on the day of admission the AUC was 0.834 (0.735–0.934) for prediction of AKI.

## Discussion

This is the first study investigating the influence of MTH on the incidence of AKI after successful CPR using the KDIGO criteria. In our population AKI occurred less often in patients treated with MTH compared to NT. This effect was more pronounced in patients with a poor neurological outcome. There was no significant difference in severe forms of AKI (stage 3) and need for RRT. In the multivariate analysis the protective effect of MTH on the development of AKI remained significant.

Understandably, most studies have focused on hypoxic brain injury following CA, but little is known about the mechanisms that occur in the kidney leading to AKI under these conditions [3]. Several animal studies of CA have demonstrated that global ischaemia and a consecutive post-CA syndrome result in significant functional and morphological injury to the kidney [6, 7, 16–18]. Hypothermia may reduce the extent of focal damage after isolated ischaemia/reflow injury (IRI) of the kidney as shown in rat model of renal transplantation [19], but reports on the renal effects of hypothermia in CA models reflecting global ischaemic injury are lacking. Previous trials on patients comparing the effect of MTH versus NT provided conflicting results [20]. In a meta-analysis of 19 trials including patients with brain injury, OHCA or major cardiovascular surgery with cardiopulmonary bypass, the use of therapeutic hypothermia did not reduce the incidence of AKI or the need for dialysis. However, a lower target cooling temperature was associated with lower odds of AKI and hypothermia was associated with lower mortality [20].

In our study the MTH-treated population had reduced incidence of AKI compared with the NT-treated population. This was mainly related to mild forms of AKI. The difference in severe forms of AKI (AKI 3) or requirement for RRT was not statistically significant. Since the group of patients treated with MTH versus NT had significant differences in their baseline characteristics a sensitivity analysis was performed based on neurological outcome, which may represent the most sensitive parameter for the severity of hypoxic damage due to CA. The beneficial effect of MTH with respect to rates of AKI was highly significant in the patients with an unfavourable neurological outcome. Though similar in trend, the effect was less apparent in patients with a favourable neurological outcome, which may be explained by the assumption that in patients with good neurological outcome the duration of hypoxia and severity of post-CA syndrome was not pronounced enough to contribute to significant renal damage. This is also reflected by the fact that the overall rate of AKI was significantly lower in patients with good neurological outcome (i.e. 45 vs. 70%). Our results correspond well with the data published by Hasper et al. [21] reporting that AKI occurs in nearly 50% of patients with CA and patients with unfavourable neurological outcome are affected more frequently. They also showed that changes in serum creatinine observed over 24 h may contribute to outcome prediction in patients post CA.

Our findings may be in accordance with published data demonstrating that targeted temperature control in kidney donors has a statistically and clinically significant protective effect on renal-graft outcomes in recipients [22]. The relative odds of delayed graft function were 38% lower when the kidneys were donated by patients assigned to a targeted temperature of 34−35 °C than when kidneys were donated by patients assigned to a targeted temperature of 36.5−37.5 °C. Furthermore, renal grafts from expanded-criteria donors and other high-risk subgroups particularly benefited from hypothermia [22]. Though these data indicate that hypothermia may attenuate the damage conferred by IRI to the kidney, it is important to note that there is a distinct difference in the pathophysiology of isolated renal IRI and AKI in the setting of systemic injury conferred by CA and the subsequent post-CA syndrome.

In our study we used cystatin C in addition to creatinine to determine renal function during the first week and at discharge to estimate renal recovery. Creatinine with its well-known limitations in the acute setting is still the standard measure of renal function, but has limited usefulness in the early detection of AKI [23]. The serum concentration is greatly influenced by numerous non-renal factors such as body weight, race, age, gender, total body volume, drugs, muscle metabolism and protein intake [24, 25]. Therefore, it has to be considered that estimation of eGFR based on serum creatinine might overestimate renal function due to loss of muscle mass in critically ill patients [26].

Cystatin C on the other hand is a low molecular-weight cysteine proteinase produced by all nucleated cells that is freely filtered through the glomerular membrane and completely reabsorbed and metabolized by the proximal tubular cells without secretion. In contrast to creatinine, serum concentration of cystatin C is not affected by inflammation, fever and/or outside agents or by muscle mass, gender or age [27–29]. As such, it has been proposed as an early biomarker of AKI in intensive care [30, 31]. The KDIGO criteria rely on serum creatinine and urine output as indicators of renal function. As muscle metabolism is often impaired in a critically ill patient, we might underestimate the occurrence of AKI especially during MTH, when metabolic rates are decreased, but probably also under NT. Therefore we also determined that cystatin C might be more sensitive for smaller changes in renal function [32] and has been shown to be strongly significantly correlated with the histopathological grade of renal injury in an animal model of ventricular fibrillation CA [33]. So far cystatin C has not been described in the context of AKI and MTH after CPR.

In the cohort treated with MTH the whole population and the subgroup of patients with poor neurological outcome had significantly lower creatinine levels than those treated with normothermia. The same pattern was observed for cystatin C levels independent of neurological outcome. Both markers were significantly reduced at several time points within 5 days after CPR in patients treated with MTH. Whether this reflects improved renal function or an altered production rate of the markers cannot be discriminated by our approach. However, in a sub study of the HACA trial, reduced creatinine clearance was observed during MTH, whereas the respective creatinine values tended to be even lower than those of the patients treated with normothermia, indicating reduced creatinine production during MTH [8].

However, at ICU discharge creatinine and cystatin C levels were still significantly lower in patients treated with MTH. This corresponds to increased eGFRs, with cystatin C-derived eGFRs consistently lower (ca. 20%) compared to eGFRs based on serum creatinine. Since it has previously been demonstrated that creatinine production is diminished during longer ICU stays [26], the eGFRs obtained from creatinine may overestimate renal function at ICU discharge in our study. However, cystatin C-derived eGFR also remained higher at ICU discharge in the patients treated with MTH and this may reflect improved renal recovery [34, 35]. Although renal function at ICU discharge may not necessarily reflect long-term renal function, the ICU LOS in patients with good neurological outcome was 8 (IQR 9) days, which would fit into the suggested observation period to evaluate early renal recovery (7–90 days) [34, 36].

### Limitations

There are several limitations of our study. First, this is a single-centre study and allocation to MTH or NT was not by randomization but according to recommendations at the time of the study. This might have led to selection bias. However, we added MTH to the multivariate analyses and added a sensitivity analysis based on neurological outcome to reduce such a bias.

Second, we only used the KDIGO criteria based on creatinine values to determine AKI due to lack of properly recorded urinary output data. Third, we did not measure creatinine clearance at discharge. To compensate for that, we added cystatin C as an additional renal functional parameter. Fourth, muscle metabolism and production of creatinine might be impaired by lower body temperature, which might lower its usefulness in estimating renal function under treatment with MTH. There are no data about the metabolism of cystatin C during MTH. However, we saw good discrimination between MTH and NT later during the stay and at ICU discharge, when MTH had already been stopped for a prolonged time. Fifth, we have no data on long-term outcome, but we can provide data on early renal recovery by showing eGFR at ICU discharge and requirement for RRT at hospital discharge.

## Conclusions

In summary, these results indicate a moderate protective effect of MTH on renal function after cardiac arrest and possibly improved recovery from AKI. Although the effect of MTH is more pronounced in mild forms of AKI, there is still a significant effect in multivariate analysis. In this context cystatin C seems to perform well as a biomarker to determine renal function under application of MTH. Further studies are needed to confirm these results.

## Additional files

Additional file 1: Flow chart. (DOCX 34 kb)

Additional file 2: **Table S1.** Patient characteristics in patients treated with mild therapeutic hypothermia or normothermia. (DOCX 14 kb)

Additional file 3: **Figure S2.** Serum creatinine and cystatin C in patients with good or poor neurological outcome. All patients: serum creatinine (mg/dl) (a) and serum cystatin C (mg/l) (b) (mean and standard deviation) at day 0–4 and ICU discharge in patients with good or poor neurological outcome; *p < 0.05, **p < 0.01. (DOCX 78 kb)

Additional file 4: **Table S2.** Estimated GFR based on creatinine (CKD-EPI) and cystatin C (CKD-EPI) at ICU discharge in patients treated with MTH or NT, with or without AKI. (DOCX 12 kb)

## Abbreviations

AKI: Acute kidney injury
APACHE II: Acute Physiology and Chronic Health Evaluation II
AUC: Area under the curve
CA: Cardiac arrest
CKD-EPI: Chronic Kidney Disease Epidemiology Collaboration
CPC: Cerebral Performance Categories
CPR: Cardiopulmonary resuscitation
eGFR: Estimated glomerular filtration rate
ICU: Intensive care unit
IRI: Ischaemia reperfusion injury
KDIGO: Kidney Disease Improving Global Outcomes
LOS: Length of stay
MDRD: Modification of Diet in Renal Disease
MTH: Mild therapeutic hypothermia
NGAL: Neutrophil gelatinase-associated lipocalin
NT: Normothermia
OHCA: Out-of-hospital cardiac arrest
ROC: Receiver operating characteristic
ROSC: Return of spontaneous circulation
RRT: Renal replacement therapy
SOFA: Sequential Organ Failure Assessment
