# Supplementary material for: Acute kidney injury and mild therapeutic hypothermia in patients after cardiopulmonary resuscitation - a post hoc analysis of a prospective observational trial
Source: Crit Care. 2018 Jun 8;22:154. doi: 10.1186/s13054-018-2061-6 (PMC5992881; doi:10.1186/s13054-018-2061-6)
Supplement: Supplementary file 1 — Flow chart. (DOCX 34 kb) [file 13054_2018_2061_MOESM1_ESM.docx]

**Table 1 Flow chart**

152 patients were included in the original study

Additional Figures

18 patients excluded from the population due to one of the following causes:

- loss of follow up N=4
- missing values N=4
- other non-neurological causes of death N=10

134 patients were included in final analysis of the original study

N= 8 patients dropped out due to CKD in previous medical history and missing baseline creatinine

126 patients were included in final analysis of the current study

53 patients without acute kidney injury

73 patients with acute kidney injury
