# Supplementary material for: Acute kidney injury and mild therapeutic hypothermia in patients after cardiopulmonary resuscitation - a post hoc analysis of a prospective observational trial
Source: Crit Care. 2018 Jun 8;22:154. doi: 10.1186/s13054-018-2061-6 (PMC5992881; doi:10.1186/s13054-018-2061-6)
Supplement: Supplementary file 2 — Table S1. Patient characteristics in patients treated with mild therapeutic hypothermia or normothermia. (DOCX 14 kb) [file 13054_2018_2061_MOESM2_ESM.docx]

**Additional file 2**

**Table 1** Patient characteristics in patients treated with mild therapeutic hypothermia or normothermia

|  | NT (n=71) | MTH (n=55) | p value |
| --- | --- | --- | --- |
|  |  |  |  |
| Age in years, median (IQR) | 67 (21) | 60 (18) | 0.007 |
| Female, n (%) | 23 (32) | 10 (18) | ns |
| Bystander-initiated CPR, n (%) | 43 (61) | 41 (75) | ns |
| Time to ROSC > 20 min, n (%) | 41 (58) | 30 (55) | ns |
| Cardiac arrest in hospital, n (%) | 12 (17) | 2 (4) | 0.019 |
| Poor neurological outcome, n (%) | 44 (62) | 20 (36) | 0.005 |
| Shockable first monitored rhythm, n (%) | 27 (38) | 48 (87) | 0.0001 |
| Catecholamines at admission, n (%) | 48(68) | 52(95) | 0.0001 |
| Baseline creatinine, MV ± SD [mg/dl] | 1.07 ± 0.99 | 0.97 ± 0.16 | ns |
| SOFA score, median (IQR) | 10 (4) | 10 (3) | ns |
| APACHE II score, median (IQR) | 26 (8) | 23 (6) | 0.0001 |
