# Supplementary material for: Acute kidney injury and mild therapeutic hypothermia in patients after cardiopulmonary resuscitation - a post hoc analysis of a prospective observational trial
Source: Crit Care. 2018 Jun 8;22:154. doi: 10.1186/s13054-018-2061-6 (PMC5992881; doi:10.1186/s13054-018-2061-6)
Supplement: Supplementary file 3 — Figure S2. Serum creatinine and cystatin C in patients with good or poor neurological outcome. All patients: serum creatinine (mg/dl) (a) and serum cystatin C (mg/l) (b) (mean and standard deviation) at day 0–4 and ICU discharge in patients with good or poor neurological outcome; *p < 0.05, **p < 0.01. (DOCX 78 kb) [file 13054_2018_2061_MOESM3_ESM.docx]

**Additional file 3 Serum creatinine and cystatin C in patients with good or poor neurological outcome**

All patients: (a) Serum creatinine [mg/dl] and (b) serum cystatin C [mg/l] levels (mean and standard deviation) at day 0-4 and ICU discharge in patients with good or poor neurological outcome * p<0.05; ** p< 0.01.
