# Supplementary material for: Acute kidney injury and mild therapeutic hypothermia in patients after cardiopulmonary resuscitation - a post hoc analysis of a prospective observational trial
Source: Crit Care. 2018 Jun 8;22:154. doi: 10.1186/s13054-018-2061-6 (PMC5992881; doi:10.1186/s13054-018-2061-6)
Supplement: Supplementary file 4 — Table S2. Estimated GFR based on creatinine (CKD-EPI) and cystatin C (CKD-EPI) at ICU discharge in patients treated with MTH or NT, with or without AKI. (DOCX 12 kb) [file 13054_2018_2061_MOESM4_ESM.docx]

**Additional file 4**

**Table 2** Estimated GFR based on creatinine (CKD-EPI) and cystatin C (CKD-EPI) at ICU discharge in patients treated with MTH or NT, with or without AKI.

| e GFR (ml/min/1,73m2) | MTH | NT | p-value | AKI | no AKI | p-value |
| --- | --- | --- | --- | --- | --- | --- |
|  |  |  |  |  |  |  |
| Creatinine, mean (IQR) | 83 (44) | 53 (55) | 0.0001 | 44 (43) | 90 (31) | 0.0001 |
| Cystatin C, mean (IQR) | 71 (46) | 38(51) | 0.0001 | 37 (39) | 77 (33) | 0.0001 |
